# Supplementary material for: Optimization of a Green Microwave-Assisted Extraction Method to Obtain Multifunctional Extracts of Mentha sp
Source: Foods. 2023 May 18;12(10):2039. doi: 10.3390/foods12102039 (PMC10217735; doi:10.3390/foods12102039)
Supplement: Supplementary file 1 [file foods-12-02039-s001.zip › foods-2376876-supplementary.pdf]

Figure S1. Petri plates containing Muller-Hinton agar medium inoculated with *S. aureus*, grown at 37 ºC for 24 h: A and B control (in duplicate), and C and D *M. rotundifolia* MAE extract (in duplicate) obtained at 75 ºC for 17.5 minutes using different solvents (from left to right and from top to bottom: water, acetone, methanol and ethanol).

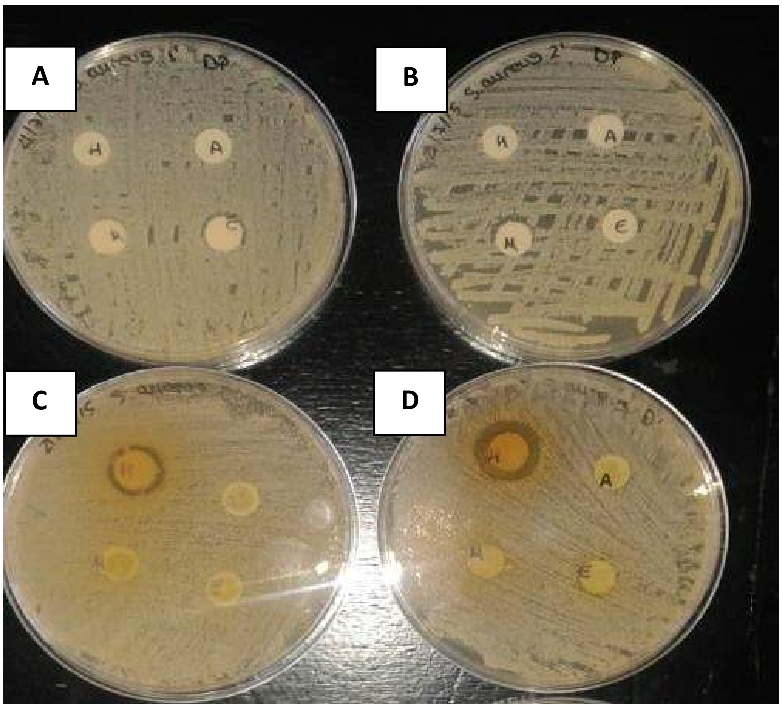

Figure S2. Evaluation of the antimicrobial activity of *M. rotundifolia* aqueous extracts obtained under different MAE conditions: A) 75 ºC, 17.5 min, B) 100 ºC, 17.5 min, and C) 100 ºC, 5 min. Petri plates containing Muller-Hinton agar medium inoculated with *S. aureus*, grown at 37 ºC for 24 h.

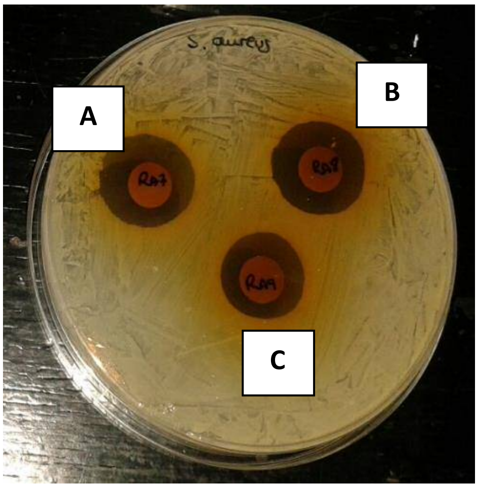

Table S1. Summary of the statistical results for ANOVA analysis of the experimental design aimed to maximize R_{TPC}.

ANOVA R_{TPC}	Sum of squares	Degrees of freedom	Mean square	F-ratio	*P*-value
T	0.98415	1	0.98415	47.45	0.0005
$T \cdot t$	0.2116	1	0.2116	10.20	0.0187
Total error	0.12445	6	0.0207417		
Total (corr)	1.3202	8			
Estimation standard error			0.14402		
Mean absolute error			0.101481		

Table S2. Summary of the statistical results for ANOVA analysis of the experimental design aimed to maximize R_{DPPH}.

ANOVA R_{DPPH}	Sum of squares	Degrees of freedom	Mean square	F-ratio	*P*-value
T	3.22667	1	3.22667	9.04	0.0299
t	1.04167	1	1.04167	2.92	0.1483
$T \cdot t$	1.3225	1	1.3225	3.71	0.1122
Total error	1.78472	5	0.356944		
Total (corr)	7.37556	8			
Estimation standard error			0.597448		
Mean absolute error			0.353086		

Table S3. Summary of the statistical results for ANOVA analysis of the experimental design aimed to maximize R_{AM}.

ANOVA R_{AM}	Sum of squares	Degrees of freedom	Mean square	F-ratio	*P*-value
T	3.68167	1	3.68167	87.20	0.0001
T^2	0.605	1	0.605	14.33	0.0091
Total error	0.253333	6	0.0422222		
Total (corr)	4.54	8			
Estimation standard error			0.20548		
Mean absolute error			0.118519		

Table S4. Compounds detected by LC-QToF MS in MAE extracts of different *Mentha* species: *M. spicata* (*MSp*), *M. pulegium* (*MP*), *M. cervina* (*MC*), *M. longifolia* (*ML*), *M. rotundifolia* (*MR*) and *M. suaveolens* (*MS*).

**Compound**	**Molecular formula**	**Chemical structure**	**Identification**				**Samples**					
			t_R (min)	[M-H]$^-$	MS/MS	Confirmation of structure	*MSp*	*MP*	*MC*	*ML*	*MR*	*MS*
Caffeic acid	$C_9H_8O_4$	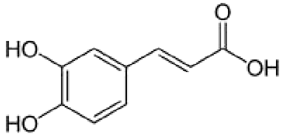	8.0	179	135/79	St, MS/MS, Lit	√	-	√	√	√	√
Caftaric acid	$C_{13}H_{12}O_9$	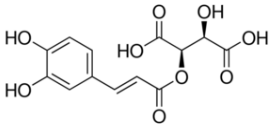	3.1	311	179/149/135/87	MS/MS, Lit	√	√	-	√	-	√
Catechin isomer	$C_{15}H_{14}O_6$	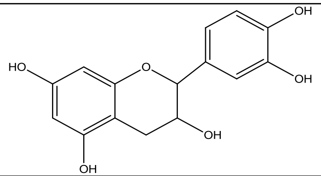	12.2	289	245/203	MS/MS	√	√	√	√	√	√
Chlorogenic acid	$C_{16}H_{18}O_9$	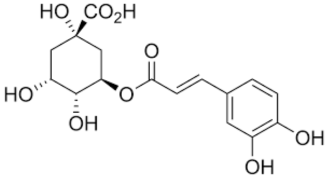	7.3	353	191/179/135	St, MS/MS, Lit	√	√	√	√	√	√
Citric acid	$C_6H_8O_7$	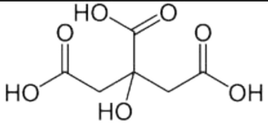	1.4	191	111/87/57	St, MS/MS, Lit	√	√	√	-	-	√

p-Coumaric acid	$C_9H_8O_3$	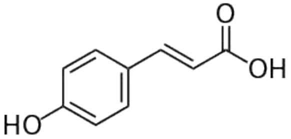	10.51	163	119/91	St, MS/MS, Lit	√	√	√	√	-	√
Cryptochlorogenic acid	$C_{16}H_{18}O_9$	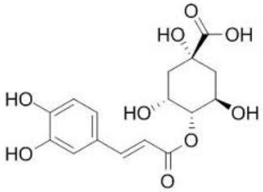	8.0	353	191/179/ 135	MS/MS, Lit	√	√	√	√	√	√
Diosmin	$C_{28}H_{32}O_{15}$	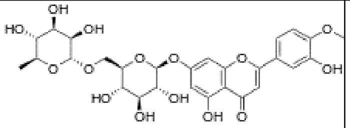	19.13	607	299	St, MS/MS, Lit	√	-	-	-	-	-
Eriocitrin	$C_{27}H_{32}O_{15}$	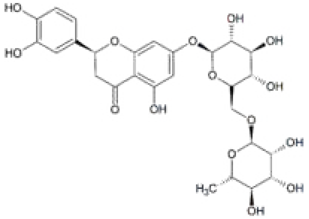	12.0	595	287/151	MS/MS	√	-	-	√	-	-
trans-Ferulic acid	$C_{10}H_{10}O_4$	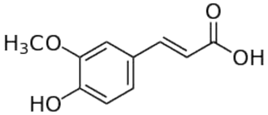	12.6	193	178/134/ 77	St, MS/MS, Lit	√	√	√	-	-	-
Gallic acid	$C_7H_6O_5$	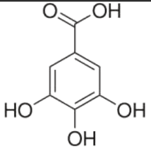	0.9	169	125/79	St, MS/MS	-	√	-	-	-	-

Gallocatechin isomer	$C_{15}H_{14}O_7$	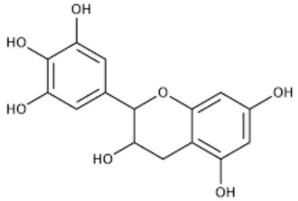	12.5	305	225/96/ 59	MS/MS, Lit	√	√	√	√	√	√
Gentisic acid	$C_7H_6O_4$	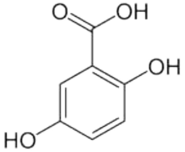	3.6	153	109	St, MS/MS	-	-	√	√	-	-
Hesperetin	$C_{16}H_{14}O_6$	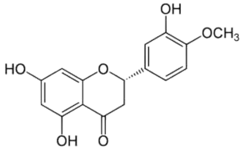	22.8	301	286/242/ 164	St, MS/MS	√	√	√	√	√	√
4-Hydroxybenzoic acid	$C_7H_6O_3$	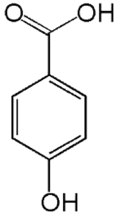	5.79	137	108 /91	St, MS/MS, Lit	-	√	√	√	√	√
5-Hydroxy-3’,4’,7-trimethoxyflavone	$C_{18}H_{16}O_6$	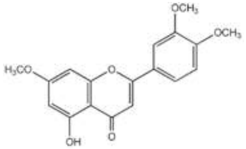	20.91	327	291/211/ 171	MS/MS	√	√	√	√	√	√

3-Hydroxy-tyrosol	$C_8H_{10}O_3$	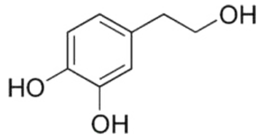	3.6	153	109/69/<br>53	St, MS/MS	-	-	√	√	-	√
Kaempferol	$C_{15}H_{10}O_6$	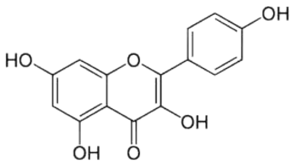	15.5	285	-	St, MS/MS	-	√	-	√	√	√
Kaempferol<br>3-*O*-glucuronide	$C_{21}H_{18}O_{12}$	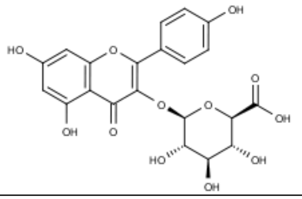	13.55	461	285	MS/MS	√	-	-	√	√	√
Kaempferol<br>3-*O*-glucuronide<br>isomer	$C_{21}H_{18}O_{12}$	--	17.10	461	285	MS/MS	√	-	-	-	√	√
Lithospermic acid<br>isomer I	$C_{27}H_{22}O_{12}$	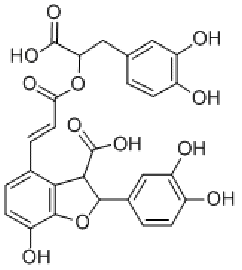	18.4	537	493/359/<br>197/161	MS/MS, Lit	√	-	-	-	-	√
Lithospermic acid<br>isomer II	$C_{27}H_{22}O_{12}$	--	19.2	537	493/359/<br>197/161	MS/MS	√	-	-	-	-	√

Lithospermic acid isomer III	$C_{27}H_{22}O_{12}$	--	19.9	537	359/197/161	MS/MS	√	-	-	-	√	√
Lithospermic acid isomer IV	$C_{27}H_{22}O_{12}$	--	20.8	537	359/197/161	MS/MS	√	-	√	-	-	√
Luteolin	$C_{15}H_{10}O_6$	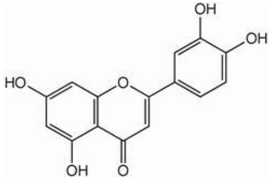	18.2	285	199/133/65	St, MS/MS	√	√	-	√	√	√
Luteolin 7-*O*-glucoside	$C_{21}H_{20}O_{11}$	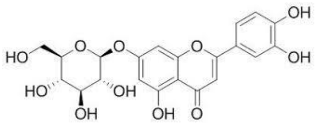	12.2	447	285	St, MS/MS	√	√	√	√	√	√
Luteolin rutinoside	$C_{27}H_{30}O_{15}$	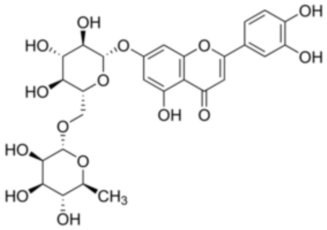	21.1	593	285	MS/MS	√	√	√	√	√	√

Neochlorogenic acid	$C_{16}H_{18}O_9$	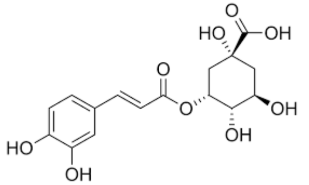	5.4	353	191/179/ 135	MS/MS, Lit	√	√	√	√	√	√
Oxalic acid	$C_2H_2O_4$	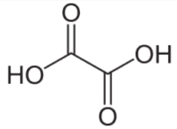	0.72	89	61/43	St, MS/MS	-	-	√	-	-	-
Quinic acid	$C_7H_{12}O_6$	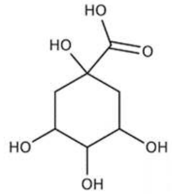	1.7	191	111/127/ 87/85	St, MS/MS, Lit	-	√	√	-	√	-
Rosmarinic acid	$C_{18}H_{16}O_8$	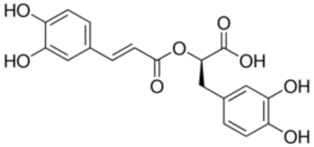	14.6	359	197/179/ 161	St, MS/MS, Lit	√	√	√	√	√	√
Rutin	$C_{27}H_{30}O_{16}$	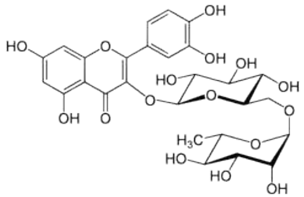	12.5	609	300	St, MS/MS, Lit	√	-	-	-	√	-

Sagerinic acid	$C_{36}H_{32}O_{16}$	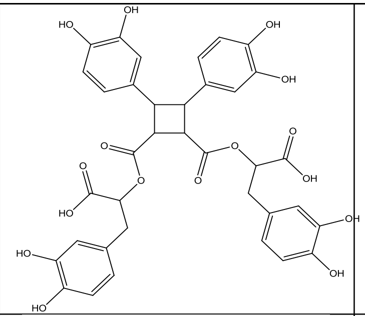	14.5	719	359/161	MS/MS, Lit	-	√	√	-	√	√
Salvianolic A acid	$C_{26}H_{22}O_{10}$	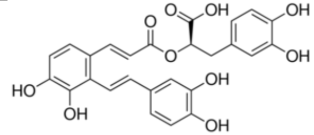	18.8	493	295/197 /161	MS/MS, Lit	√	-	-	-	√	√
Salvianolic A acid isomer	$C_{26}H_{22}O_{10}$	--	20.1	493	295/197/ 161	MS/MS	√	√	√	-	√	√
Salvianolic acid B	$C_{36}H_{30}O_{16}$	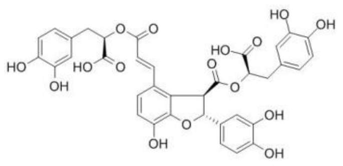	17.8	717	519/339/ 321	St, MS/MS, Lit	√	-	-	-	√	-
Salvianolic acid E	$C_{36}H_{30}O_{16}$	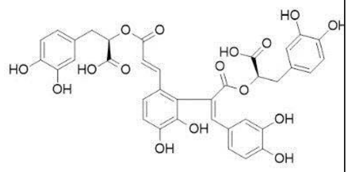	16.6	717	519/321	MS/MS, Lit	√	-	√	-	-	-
Salvianolic acid L	$C_{36}H_{30}O_{16}$	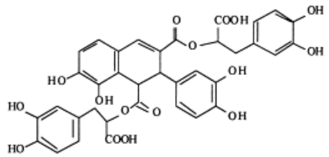	18.4	717	519/321	MS/MS, Lit	√	√	√	√	√	-

Syringic acid	$C_9H_{10}O_5$	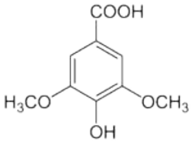	2.2	197	179/135/72	St, MS/MS, Lit	√	-	√	√	√	-
Vanillic acid	$C_8H_8O_4$	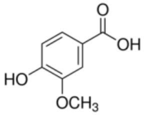	7.6	167	152/108	St, MS/MS, Lit	-	√	√	√	-	-

√: presence, -: absence. St: commercial standard available; MS/MS: confirmation by tandem mass spectrometry; Lit: data from literature [27,28,30-32] or from Metlin database.
